# A Responsive and Precise Particle Position Control System Combining a Sidewall-Driven Peristaltic Micropump and a High-Speed Camera

**DOI:** 10.3390/mi17020147

**Published:** 2026-01-23

**Authors:** Yuta Tanaka, Toshio Takayama

**Affiliations:** Department of Mechanical Engineering, Institute of Science Tokyo, 2-12-1 Ookayama, Meguro-ku, Tokyo 152-8552, Japan

**Keywords:** microfluidic device, micropump, sidewall-driven, position control

## Abstract

The systems to manipulate a single particle in a microfluidic channel can be adopted to pharmacological and cytological experiments of single-cell observation. The common cell position systems use syringe pumps driven by piezoelectric devices, and these have a flow quantity limit. To achieve single-cell manipulation using actuators without limiting the flow quantity and with a low risk of contamination, we propose a particle control system that uses a sidewall-driven peristaltic micropump driven by pneumatic pressure. The adopted pump was integrated into a single-layer mold with a flow path and was simple to fabricate. Unlike syringe pumps, it not only pumps water forward, but also inhales from the back simultaneously, and can pump indefinitely. We developed a responsive and precise particle position control system using this pump in combination with a high-speed camera. In this system, the pumping pressure is operated by real-time adjustment of a pneumatic pressure supply to realize PID control. This approach moves the particle rapidly when it is far from a designated target position for a quick approach and slowly near the target position to position precisely.

## 1. Introduction

### 1.1. The Research Background of the Pumps for Microfluidic Devices

Microfluidic devices are designed to control the flow of fluids based on their microscopic structures. They are utilized in biochemistry experiments, and are economical in terms of time, amount of reagents, and space for the reaction. In addition, studies have noted that experiments including the replication of small structures of creatures [1] can be realized with such a design. Pumps are significant elements for microfluidic devices to control fluids. There are previous studies of pumps to control fluids in microfluidic devices.

### 1.2. Previous Studies of Pumping Systems

#### 1.2.1. Single-Directional Pumps

In an example from a previous study, a system generated a flow with the asymmetrical flow resistance of conical tubes [2]. Flow direction is determined by the direction in which the conical tube is installed.

A few systems have also used centrifugal force to operate reagents. In an infection diagnostic system, centrifugal actuation has been applied to compose a high-throughput testing system by integrating multiple functions such as reagent storage, sedimentation, filtration, valving, decating, aliquoting, mixing, separation, serial dilution, and washing [3]. In a drug-screening system, various combinations of detachable inner disks and outer rings can realize flexible experimental configurations by applying a modular design approach [4].

Furthermore, some systems were developed to regulate flow with adjustable valves. A previous study utilized thermal expansion of elastomers caused by heating with microheaters to close off the relevant channel sizes [5]. This valve was as responsive as 0.1 to 0.3 s
 on one-directional flow velocity control. Microvalves made of light-driven gel have also been developed [6]. These actuators are designed to control the speed of one-directional flow.

#### 1.2.2. Bidirectional Pumps

Electro-osmotic pumps and Electrohydrodynamic (EHD) pumps are used to control the flow velocity along the micro-flow paths [7,8]. These pumps can control flow velocity in a straightforward manner with the voltage. They can change the flow direction by switching the applied voltage direction.

Syringe pumps and diaphragm pumps are also used to control microfluidic flow [9,10,11]. These pumps generate flow by pushing fluid out of a space and generate flow in the opposite direction by drawing it in.

Peristaltic pumps are also used to control microfluidic flow [12,13,14,15,16,17,18]. These pumps generate flow with progressive wave deformation of the wall of the flow path. They can change the flow direction by switching the direction of wave motion.

### 1.3. Existing Cell Manipulation Systems Using Pumps

Currently, various experiments have been conducted using this technology for microscale single-cell manipulation [19,20,21,22,23,24]. We believe that a cell-positioning system that can be produced easily and can operate for a long time is desirable for the development of this field of experimentation. We planned to manipulate cells using the fluid flow generated by pumps. The pumps are required to correct the positioning error in both directions of the linear flow path. Therefore, the pumps designed to generate or control one-directional flow are not suitable for this purpose.

There are cell manipulation systems using electro-osmotic pumps. When used to manipulate cells, it is possible to avoid cell damage due to the electric field generated by the pumps by isolating the cells from the pumps [25]. However, a special remedy for the presence of ions generated by pump electrolysis is required. Although sealing the electrode and flow path with a membrane or gel can prevent or delay the inflow of ions, additional complications like this are desirable to avoid.

A particle control system with a pseudopotential well generated by a controlled laminar flow has also been developed [26]. The purpose of this system is to manipulate a particle within an intersection of a flow path. Single particles were trapped at the stagnation point of a planar extensional flow from the opposing flow paths of the intersection, generated using a simple cross-slot channel design with two on-chip valves to precisely control the flow rate with particle position feedback to enable particle trapping and manipulation. This system achieved a precision of 1 μm on both the horizontal and vertical axes. This technology was developed specifically to control the particles on the flow stagnation points using unidirectional flow in four channels. While control is in progress, continuously inflowing from two opposing channels and continuously discharging from the remaining two channels of an intersection. Therefore, it is impossible to control the particles along a linear flow path without a branch because the particles in this system cannot remain in the straight flow path.

In addition, syringe pumps or diaphragm pumps driven by piezoelectric devices have been used for particle position control. Piezoelectric devices are responsive and can move with fine resolution and precision. Therefore, key experiments have been conducted to test the deformability of red blood cells by inserting the cells into a narrowed section set on the flow path for a highly controlled interval [9]. Such systems have a flow quantity limit because of the shortness of the motion stroke of the piezoelectric devices. As noted in reference [10], the stroke of piezoelectric devices can fail to compensate for the accumulation of positioning error, and performing a continuous positioning experiment for a long period of time is difficult. In another study, this problem was resolved by combining the system described above with other actuators that could pump relatively large quantities of water, such as gravity-driven actuators [10] and micrometer calipers [11]. However, it is more complicated to control the particle position with two actuators operating in parallel rather than using only a single actuator. Micropumps with repeated movements can be used to realize a simple system independently because they can discharge fluids without a quantity limit.

### 1.4. Peristaltic Pumps

In this study, we developed a visual feedback particle-manipulation system using sidewall-driven peristaltic micropumps. Peristaltic pumps generate flow with progressive wave deformation of the wall of the flow path, and contamination or generation of ions is avoided. Furthermore, these pumps not only pump fluids forward but also simultaneously inhale from the back, and can pump limitlessly. By switching the order of the peristaltic motion, these pumps can change the flow direction and can be adopted for particle position control. Some studies have been conducted on the operation of microfluidic flow with peristaltic pumps. In a previous study, researchers prevented contamination and used external peristaltic pumps [12,13]. However, pumps placed inside microdevices are hypothetically more responsive than external pumps because they are closer to the control region and are less affected by the flow resistance. Therefore, microfluidic devices that contain pumping structures have been developed. An optimal design for operation using the fewest pneumatic supply tubes was proposed [14]. This pump has two layers, and the pressurizing chambers placed on one layer cause the deflection of the membrane separating layers into flow paths placed on the other layer to operate the flow velocity. However, fabricating multilayered microfluidic devices is time-consuming and costly because it requires the production of molds for every layer and precise relative positioning between the layers when bonding them. On the other hand, single-layer devices require only one mold and no positioning during bonding. Therefore, pumping structures comprising a single layer are easier to fabricate than multiple layers. The sidewall-driven micropump adopted in our system can be fabricated from a single-layered mold that is integrated with pumps and flow paths. It consists of a flow channel with drive chambers installed on both sides. The walls between the chambers and the flow channel are deformed to narrow the flow path when the internal pressures of the chambers increase. The pressure is supplied from the compressed air stored in the tank, and the pressurizing chambers are switched by the three-port solenoid valves. Previous research has clarified that periodic deformation causes the flow of water inside the flow channel [15].

Peristaltic pumps generate intermittent flows. The pulsation of peristaltic pumps has been effectively utilized for droplet generation. A study demonstrated a droplet generator by operating two pumps that pumped different immiscible fluids, in a reverse-pulse manner, and two immiscible fluids were pumped alternately to form droplets [16]. However, when peristaltic pumps are used for particle position control, pulsation can cause a disturbance that should be reduced. In one study, placing air dampers on flow paths was proven to be effective for pulsation reduction [17]. In addition, flexible tubing between microfluidic devices and pumps has also been shown to be effective [18]. However, the phase delay of the damper’s flow transfer may deteriorate the responsivity of the position control system. Therefore, pulsation reduction without phase delay is desirable. It has been proven that pulsation is reduced by positioning micropumps on both ends of the flow channel and operating them in the reverse phase because of the resistance provided by one pump for the backflow of the other pump [15].

We developed a position-control system for particles floating inside a flow channel by combining peristaltic micropumps and a high-speed camera. By adjusting the pumping pressure, a smooth transition from a fast approach to the target position to a slow movement for precise positioning was realized. Using this system, experiments to control the position of a polymer microbead with a diameter of approximately 4.5 μm resulted in the successful positioning of the particle’s center of gravity within a 1.5 μm range of the target position, with an average time of approximately 0.5 s
.

### 1.5. Comparison with Previous Studies

The most common method for precise position control is to pump fluids with syringe pumps driven by piezoelectric pumps. Table 1 shows a comparison of peristaltic pumps and piezoelectric-driven syringe pumps. Whereas they can precisely control flow rate, peristaltic pumps exhibit fluid pulsation in accordance with the driving cycle. Therefore, peristaltic pumps require pulsation reduction remedies to position particles precisely [15,17,18]. On the other hand, syringe pumps driven by piezoelectric devices saturate after a certain point due to the short stroke of piezoelectric actuators [10], whereas peristaltic pumps can pump indefinitely because they not only pump water forward, but also inhale from the back simultaneously. Therefore, peristaltic pumps are more suitable for long-term positioning experiments than syringe pumps driven by piezoelectric devices.

In previous sidewall-driven peristaltic pumps, the step width of the flow could not be varied. Therefore, they cannot be used for high-precision positioning. However, we developed a pumping system using sidewall-driven peristaltic pumps that can adjust the step width by changing the pneumatic pressure supply. Thus, we realized precise particle control using the proportional–integral–derivative (PID) method.

## 2. Materials and Methods

### 2.1. Operating Principle and Channel Design

**Figure 1:**
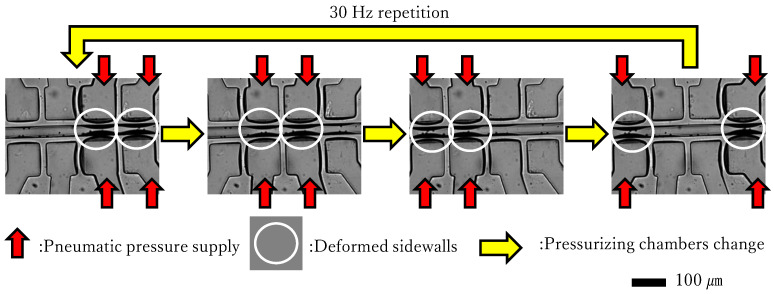
Motion of the pump.

As mentioned in the Introduction, sidewall-driven peristaltic micropumps pump water inside the flow channel with a repeated narrowing motion of the sidewall of the flow path. The pumping motion is illustrated in Figure 1. A sidewall-driven micropump consists of a flow channel in which water flows along small particles, along with four pairs of chambers. The tubes are connected to these chambers, and air pressure can be applied to the chambers through the tubes. The red arrows represent this pressure supply. When the internal pressure of the chambers increase, the walls between the flow channel and the chambers, which are enclosed by a white oval in Figure 1, deform to narrow the flow channel. This narrowing motion directly affects the flow of water inside the flow channel. By switching pressurizing chambers in the order indicated by the yellow arrows in Figure 1, peristaltic motion is induced. The compressed air stored in the tank supplies pressure, and the three-port solenoid valves switch the pressurizing chambers.

In previous research, pressurizing at a 0.5 duty ratio, 30 Hz
 frequency proved to be the best configuration, as shown in Figure 1 [15]. Gases can pass through a thin Polydimethylsiloxane (PDMS) membrane [27]; thus, bubbles can intrude into the flow channel in the presence of gas inside the chamber. To prevent this phenomenon, we filled the chambers with water beforehand, transferred the pressure from the compressed air to the wall, and deformed it. The direction of the flow can be changed by switching the pressurizing order. We hypothesized that a particle inside the flow channel can be moved to a designated position by switching the direction depending on the particle’s position relative to the target position. However, although a particle moves close to the target position, precision is low, and it moves back and forth around the destination. As the particle is moved in steps by the peristaltic pumps, a reduction in the step width is necessary to improve the control precision. The positioning error was successfully reduced by decreasing the air pressure supply to narrow the step width when the particle moved near the target position [28].

The pumps adopted in previous research took time to position the particles owing to their weakness in dispensing pressure. We assumed that extending the pump chamber length would improve the pump pressure. To optimize the length of the pumps, we measured the dispensing pressure of pumps with different lengths and selected the appropriate length.

### 2.2. Microchip Fabrication

The device mold was fabricated according to the process specified in [29]. A Si wafer was washed with isopropyl alcohol and hydrophilized using a hydrophilic treatment device (PIB-10; Vacuum Device Inc., Ibaraki, Japan). It was spin-coated with SU8-3050 (KAYAKU Advanced Materials, Westborough, MA, USA) at a target height of 100 μ
m
 and pre-baked at 95 °C for 45 min
. SU8 cells were ultraviolet(UV)-irradiated using a maskless exposure system(PALET; NEOARK Corporation, Tokyo, Japan) in a pattern designed beforehand. After post-baking at 95 °C for 5 min
, the Si wafer was agitated inside a bin thinner to develop the designed pattern.

PDMS (SILPOT184; Dow, Midland, MI, USA; base:curing agent = 9:1 (mass ratio)) was poured into the mold fabricated using the above process and cured to transfer the developed pattern. The surfaces of the processed PDMS and glass slides were subjected to hydrophilic treatment and bonded to each other to form a flow path.

### 2.3. Feedback Principle of Particle Position

Although this system was developed for biological particle samples, such as cells, we used polystyrene particles instead of them, considering the material cost. We assumed that microbeads whose diameter is from 1 μ
m
 to 10 μ
m
 are appropriate to substitute for blood cells. We used 4.5 μ
m
 microbeads (Polybead Polystyrene 4.5 Microspheres; Polysciences, Philadelphia, PA, USA) for position control, which was achieved through visual feedback using a high-speed camera (CHU130EX; SHODENSHA, Tokyo, Japan). The microbeads were then dispersed in pure water. The density of the microbeads was sufficiently close to pure water to ignore the effects of gravity on their movement.

Position control was implemented by repeating the following four processes.

1. Acquiring the image of the flow path.
2. Identifying pixels that displayed particles.
3. Deriving the position of the center of gravity of a particle.
4. Feedback to the pump operation.

The four processes above were designed to be executed in parallel by a computer.

In process 1, the PC acquired the 256-level grayscale image of 640
×
480
 pixels corresponding to the area of 300
μ
m
×
225
μ
m
 on the flow path at the rate of 150 fps.

In process 2, the pixels displaying the particle were identified. When a pixel captures part of the particle, it becomes darker. Therefore, the pixels that become darker than in the original condition can be identified as the pixels displaying the particle. An image that displays the flow path is stored beforehand and referred to as the original condition. Two remedies were implemented to avoid unintentionally capturing the noise. First, image flow path images were taken at 150 fps for 0.5 s
, and for each pixel, the lowest value was stored as the reference frame. Second, a certain threshold was set, and the fluctuation in the measurement acquired by a high-speed camera with an amplitude smaller than the threshold was ignored. However, if the threshold is set too high, particle capture fails owing to a lack of sensitivity. Therefore, considering the tradeoff between sensitivity and noise reduction, we set 50% as the threshold. Therefore, binarization was conducted as follows: If a pixel had a brightness of less than 50% of the corresponding pixel in the image captured in advance, it was identified as a pixel displaying the particle. By contrast, if the brightness was equal to or greater than 50% of the brightness in the comparison, it was regarded as a pixel displaying the background. By executing the categorization for each pixel, they were classified as “particles” and “background.”

Process 3 was conducted to calculate the position of the center of gravity of a particle by executing the following procedures.

**Figure 2:**
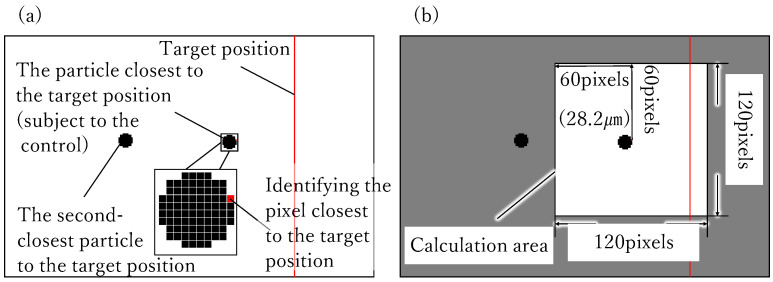
(**a**) Procedure 3.1. of center of gravity calculation. Identifying the closest pixel to the he target position indicated by the vertical red line in the figure. (**b**) The procedure 3.2. Calculating to derive the center of the gravity of the particle within the area around the pixel identified in (1).

3.1. Identifying the pixel closest to the target position from the ones categorized as “particles” in the binarization process (Figure 2a).
3.2. Deriving the center of the gravity in the region of 120
×
120
 pixels around the pixel identified in process 3.1. (Figure 2b).

The region used to calculate the center of gravity was narrowed to limit the subject to a single particle. The center of gravity was derived using Equation (1). The variables used in Equation (1) are listed in Table 2. Reference [30] states that the error in the calculated value of the center of gravity using Equation (1) from the real value is dependent on the diameter of the particle. Assuming that the particle is captured as a circle with a diameter of 4.5 μ
m
, the RMSE is less than 0.07 μ
m
 (0.15 pixels).

(1) x
C
o
G
=
i
C
o
G
D
=
∑
i
∑
j
w
(
i
,
j
)
·
i
∑
i
∑
j
w
(
i
,
j
)
·
1
D

### 2.4. Pump Operation Principle

**Figure 3:**
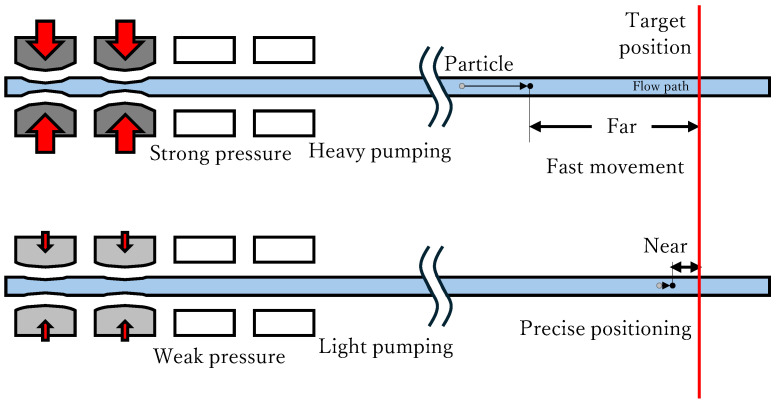
Schematic diagram of the particle position control system: When the particle is far from the target position, it is moved fast to approach the target position quickly by deforming the pump chambers largely and pumping heavily. On the contrary, when the particle is near the target position, it is moved slowly to position precisely by deforming the pump chambers slightly and pumping lightly.

Responsiveness and precision are required to adopt the particle control system for the experiment. Moving the particles slowly is effective in achieving precise positioning. However, they take quite some time to position when moving slowly, and responsiveness is sacrificed. Therefore, we developed a system that can move a particle quickly when it is far from the target position and slowly when it is near the target position, as shown in Figure 3. We assumed that the particle speed could be controlled by adjusting the deformation of the pump chambers. In the system we developed, strong pneumatic pressure was supplied to the pump when the particle was far from the target position and reduced as the particle approached the target position; therefore, the deformation of the pump chambers was adjusted, and the particle slowed down as it approached the target position. The amplitude of the pneumatic pressure supply was controlled using an electropneumatic regulator. To realize the transition from fast movement to slow positioning, as described above, the control input was determined to be proportional to the positioning error. Integral and derivative controls are introduced to compensate for static disturbance and reduce overshoot, respectively. Therefore, we adopted the PID control method to compose the system. Thus, we systematically developed a PID particle manipulation system based on a high-speed camera, three-port solenoid valves, and an electropneumatic regulator using a computer program.

**Figure 4:**
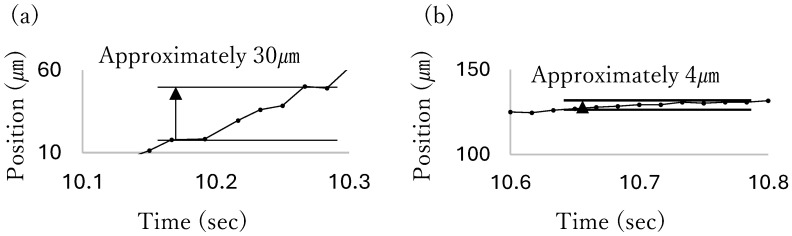
Comparison of the movements of the particle on the chip with the height approximately 100 μ
m
, the width 30 μ
m
, and chamber length 100 μ
m
. (**a**) The movement when the pump was driven with 0.3 MPa
. (**b**) The movement when the pump was driven with 0.1 MPa
 (excerpt from the research data in reference [15]).

Figure 4a illustrates the movement of a particle when the pump, 100 μ
m
 in pump chamber length, approximately 100 μ
m
 in height, 30 μ
m
 in width, was driven by an air pressure of 0.3 MPa
 and a repeating frequency of 30 Hz
. The particles then moved in steps. Figure 4b shows the particle movement when the same pump was driven with 0.1 MPa
 air pressure. This indicates that the step length decreased when the pressure supplement decreased [15]. Therefore, to position the particle as quickly and accurately as possible, it is hypothetically effective to move the particle quickly in large steps by driving pumps with high pressure when the particle is far from the target position, and then reduce the pressure when the particle approaches the destination to position precisely through fine steps. Therefore, a control program was designed to make the pumping pressure proportional to the deviation of the particle position from the target position. The air supply was adjusted using an electropneumatic regulator (ITV2030; SMC Corporation, Tokyo, Japan). The relationship between the supplied pneumatic pressure and pumping power was measured beforehand, and the supplied pneumatic pressure was operated to make the pumping power proportional to the control input.

## 3. Results

### 3.1. Pump Design to Bolster Power

**Figure 5:**
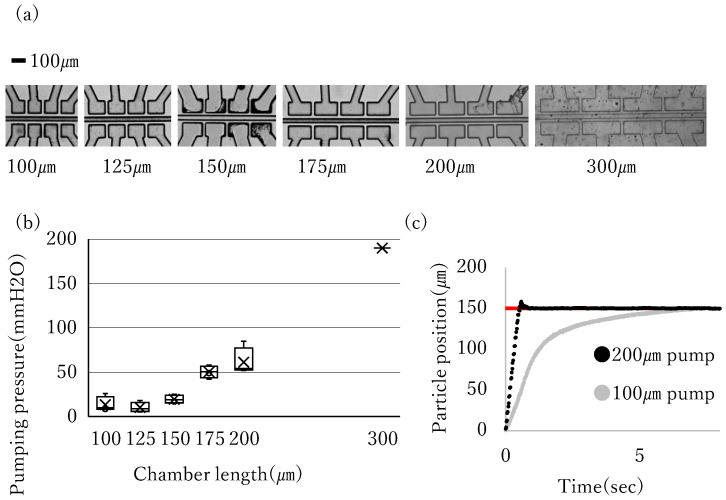
Experiment to clarify the relationships between the chamber length and the pumping power. (**a**) The photograph of the pumps of different sizes. (**b**) Box-and-whisker plot created based on results obtained by supplying 0.35 MPa
 of air pressure to the pump and measuring the discharge pressure. (**c**) The comparison of the step response between the pumps of different sizes. The red line indicates the target position.

**Figure 6:**
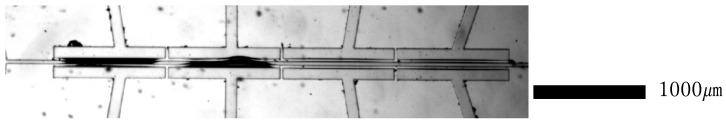
Flow channel, which was broken in the process of fabrication.

It is necessary to enhance the pumping pressure to expedite positioning. We assumed that the pumping pressure can be increased by lengthening the pump chamber. Pumps with different longitudinal dimensions were tested to test this hypothesis. Firstly, we fabricated pumps with longitudinal dimensions of the pump chamber in increments of 25 μ
m
 from 100 μ
m
 to 200 μ
m
, and the discharge pressures were measured four times for each pump. The measured pumping pressures for each variation in the chamber length driven at 0.35 MPa
 are shown in Figure 5b. As shown in this graph, the pumping power was greater for a longer pump chamber. In a previous study [28], positioning response comparison between 100 μ
m
 and 200 μ
m
 pumps was conducted. As shown in Figure 5c, 200 μ
m
 pumps were apparently responsive than 100 μ
m
 ones. Therefore, we decided to fabricate a longer pump than 200 μ
m
 to realize better responsivity. However, it was not possible to create pumps of any length. When the designed pump was too long, it could not be fabricated because of the lack of rigidity of the chamber walls. For example, we failed to fabricate a pump with the designed chamber length of 1000 μ
m
 and the flow path collapsed (Figure 6). This fact indicates that 1000 μ
m
 is beyond the upper limit of chamber length we can produce steadily. Given that the fabrication success rate is important as well as positioning responsivity, we decided to adopt pumps shorter than 1000 μ
m
. We found that pumps with chambers 300 μ
m
 in length could be fabricated steadily. Considering the tendency indicated in the experimental data from 100 to 200 μ
m
 pumps, it could be assumed that 300 μ
m
 pumps can pump more strongly than shorter ones. Pumping pressure of 300 μ
m
 pump chamber with 0.35 MPa
 measured one time is also included in Figure 5c. Though it was measured only once, higher pumping pressure than 200 μ
m
 was measured consistently with the hypothesis that a longer pump can pump stronger. Although it has yet to be proven optimal, 300 μ
m
 pumps were adopted as the design dimension.

### 3.2. Estimated Flow Rate

We decided to evaluate the flow rate the pump can handle quantitatively. However, since the flow rate is extremely low, we determined that direct measurement would be difficult. Therefore, we experimentally measured the velocity distribution across the flow channel cross-section. We then approximated the flow rate by integrating over the cross-section using existing theoretical values for the velocity distribution.

**Figure 7:**
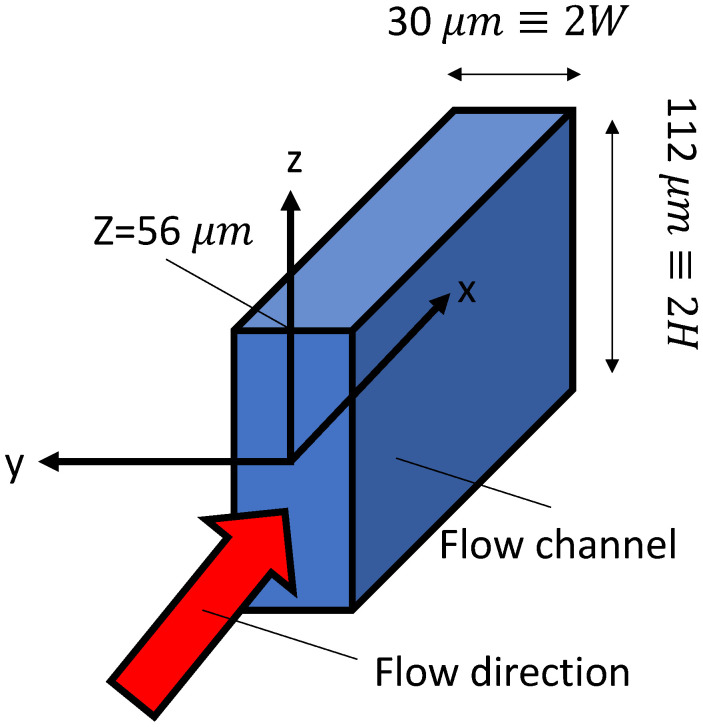
Coordinate system.

The coordinate system defined relative to the flow channel is shown in Figure 7. The origin is placed at the center of the flow channel cross-section, with the x-axis in the flow velocity direction, the y-axis in the flow channel width direction, and the z-axis in the flow channel height direction. The flow channel height is 112 μ
m
 ≡ 2H, and the flow channel width is 30 μ
m
 ≡ 2W.

According to Adachi, the velocity distribution in a rectangular channel is expressed by Equation (2) [31].

(2) U
(
y
,
z
)
=
1
−
y
2
−
r
∑
n
=
0
∞
(
−
1
)
n
cos
m
y
cosh
m
z
m
3
cosh
m
A
w
h
e
r
e
A
≡
H
W
,
m
≡
2
n
+
1
2
π

**Figure 8:**
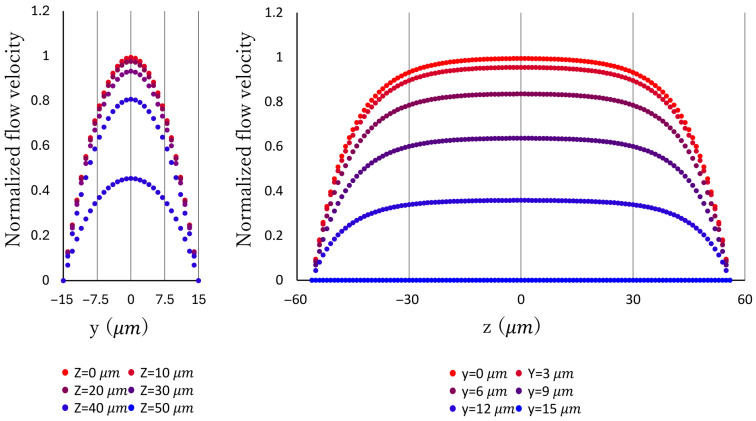
Theoretical value of flow speed distribution inside a rectangular flow channel.

Note that *U*, *y*, and *z* in Equation (2) are dimensionless quantities based on the channel center velocity U
0
 and with *W*. An approximate equation derived from this formula was used to estimate the actual velocity distribution across the cross-section from measured data. The velocity distribution is shown in Figure 8.

**Figure 9:**
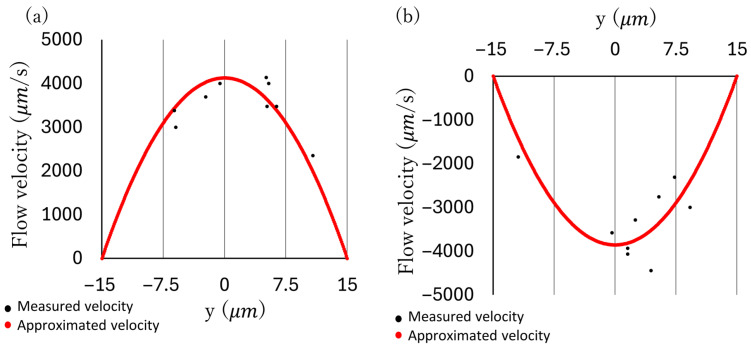
Flow rate approximation (**a**) Measured data and approximated values of flow velocity when pumping forward, where (z = 0). (**b**) Measured data and approximated values of flow velocity when pumping backward, where (z = 0).

To measure the flow velocity distribution, a suspension of microbeads was pumped at maximum discharge pressure. The relationship between the position of the microbeads on the cross-section and their velocity was measured using a high-speed camera. The focus of the high-speed camera was set near z = 0, and only particles in focus were measured. The flow velocity U
0
 at the channel center was approximated using the gradient method to minimize the sum of squared errors with the measured values, thereby deriving the flow velocity distribution at z = 0. Figure 9a,b show graphs overlaying the approximated and measured values for the forward and reverse directions, respectively. Having obtained the velocity distribution at z = 0, the flow rate was calculated by integrating the velocity distribution predicted by Equation (2) over the entire channel cross-section based on this distribution. The derived values were 7.7
×
10
6
μ
m
3
/
s
 in the positive direction and −
7.2
×
10
6
μ
m
3
/
s
 in negative direction.

### 3.3. Determination of the Pneumatic Pressure Supply Algorithm

**Figure 10:**
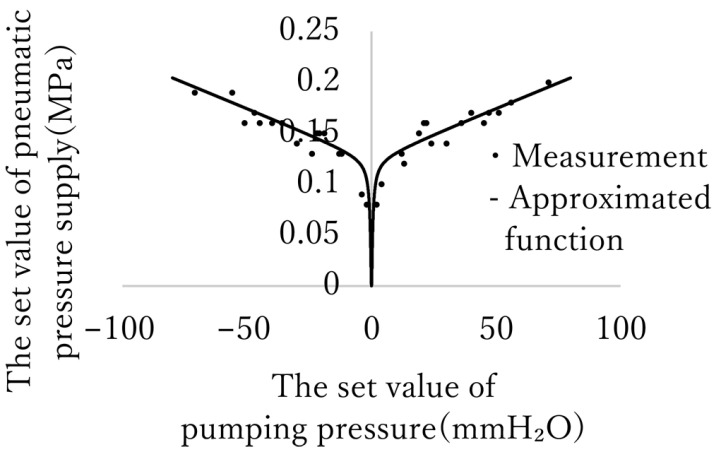
Measured pumping pressure and approximated function to set the pneumatic pressure supply. The sign on the pumping pressure corresponds to its direction.

The relationship between the supplied pneumatic pressure and dispensing pressure was measured. The results are shown in Figure 10. These data were approximated using Equation (3), which was then used to derive the set value of the pneumatic pressure supply.

(3) P
:
Set
pneumatic
pressure
supply
(
MPa
)
u
:
Set
pumping
pressure
(
m
m
H
2
O
)
P
=
0.11
×
arctan
(
2.3
×
u
)
−
0.03
×
arctan
(
8.33
×
u
)
+
0.001
×
u

### 3.4. Step Response Measurement

**Figure 11:**
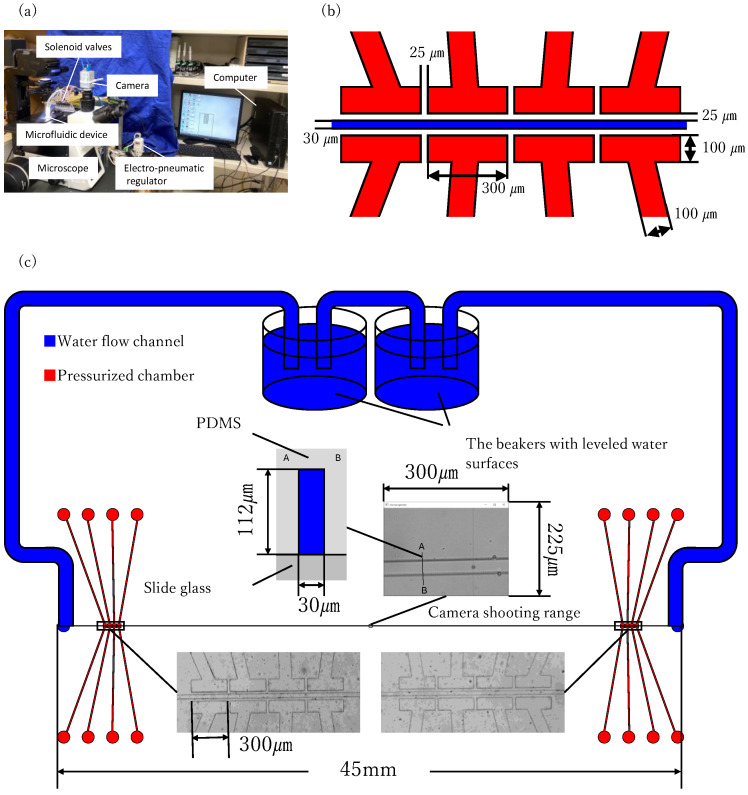
(**a**) Photograph of the entire system (**b**) Dimensional drawing of the device (**c**) Schematic diagram of the entire system.

Figure 11 shows a schematic of the overall system. The height of the mold was approximately 112 μ
m
, the flow channel width was 30 μ
m
, and the length of the pump chambers was 300 μ
m
. The ends of the flow path were connected to beakers with leveled water surfaces.

**Figure 12:**
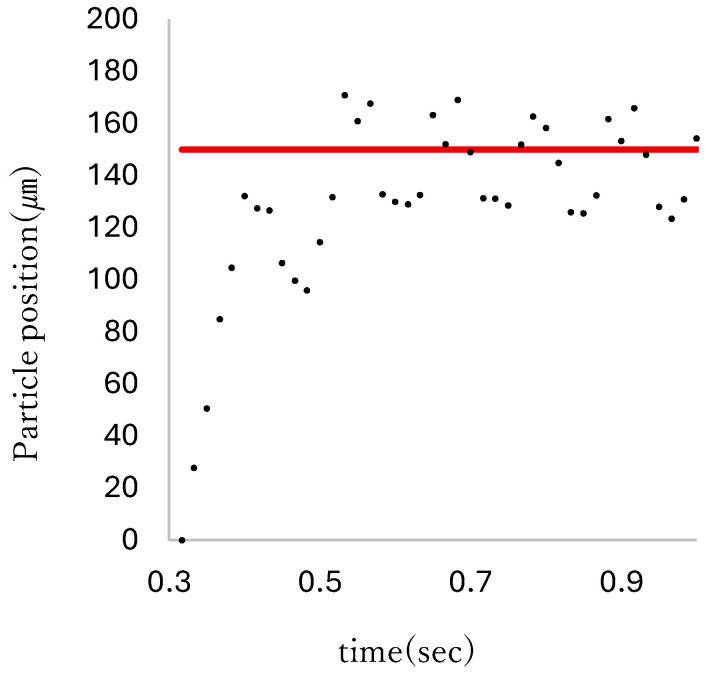
Particle oscillation. The red line indicates the target position.

When the pneumatic pressure supply was determined without using Equation (3), the particle oscillated around the target position. Figure 12 shows a plot of the particle motion during this phenomenon. To control the particles precisely and quickly, it was necessary to adjust the proportional gain between the pumping pressure and the pressure supply.

**Figure 13:**
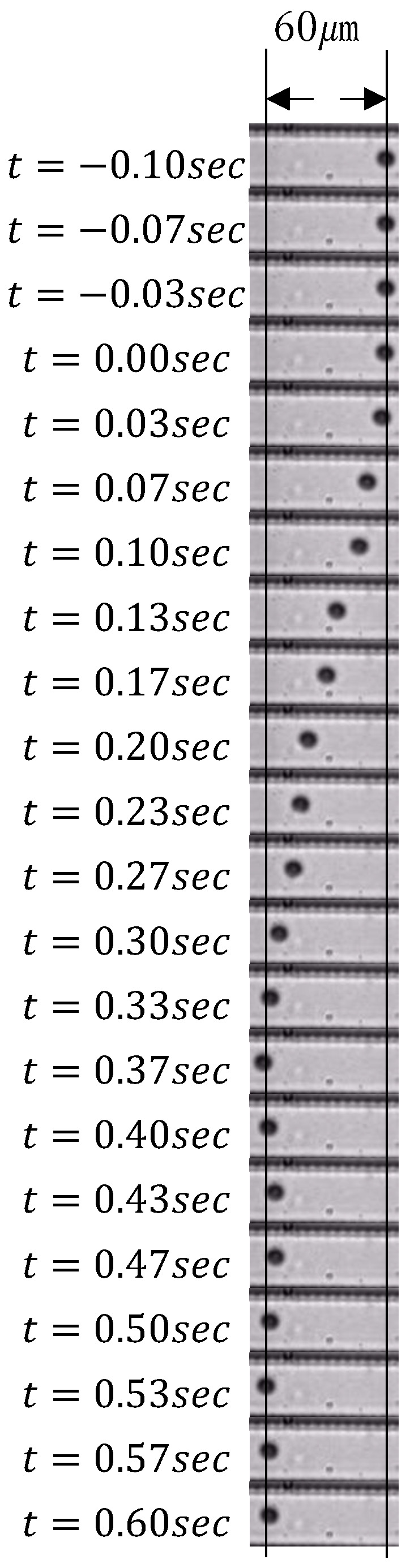
Sequence of images of a response.

Sixty μm step responses were recorded for 20 s, ten times to evaluate the positioning speed and precision. Figure 13 shows a sequence of images from one of the recorded responses.

### 3.5. Evaluation of Step Response

**Figure 14:**
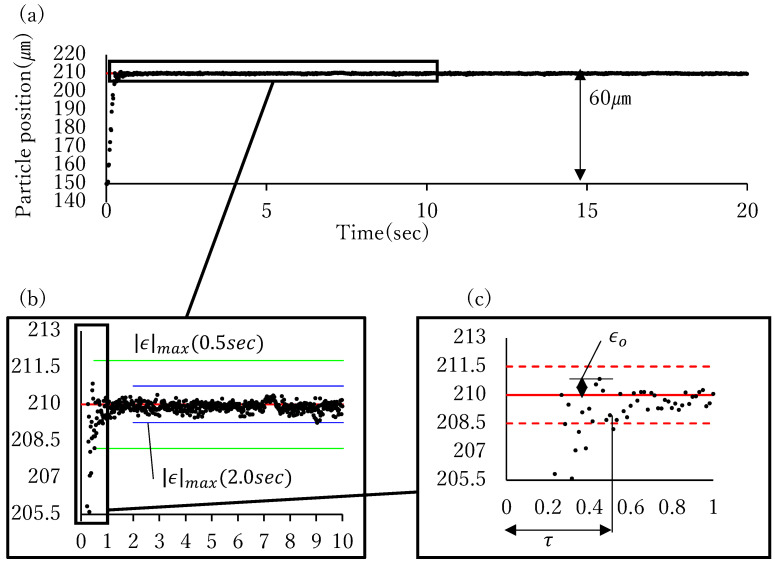
(**a**) Particle motion in step response. (**b**) Definition of maximum absolute error. Two values of the maximum absolute error were adopted: one was calculated using data from 0.5 s
 after the step input, and the other was calculated from data from 2.0 s
 after the step input, which are indicated as green and blue lines in this Figure, respectively. (**c**) Definition of positioning time and overshoot. Positioning time is the time taken to position within the region enclosed by red dashed lines.

**Figure 15:**
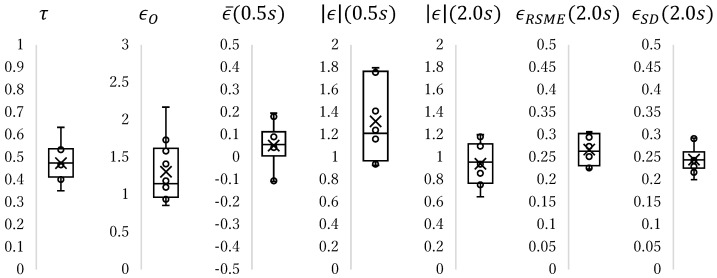
Box-and-Whisker plot showing experimental result of step response.

The overshoot, time taken to reach the position, maximum absolute error, mean error, root mean squared error (RMSE), and standard deviation were used to assess the positioning speed and precision. The definitions of these variables are presented in Table 3. The positioning time is defined as the time from the step input time to the time when the last position enters the region within 1.5 μm of the target position (the region enclosed by the red dashed line in Figure 14c). Two values of the maximum absolute error were adopted: one was calculated using data from 0.5 s after the step input, and the other was calculated from data from 2.0 s after the step input (green and blue lines in Figure 14b, respectively). The mean error, RMSE, and standard deviation were calculated using data from 0.5 s after the step input was adopted. The measured values of each item are shown in Figure 15. It took 0.47 s on average, precision from 0.5 s after input was 1.32 μm and from 2.0 s was 0.94 μm.

## 4. Discussion

### 4.1. Evaluation of Particle Positioning

Although it is standard to evaluate flow velocity when it comes to micropumps, we concentrated on evaluating particle movement because the primary subject of this study was particle position control. As shown in Figure 13, setting an overly large proportional gain between the pumping power and particle position prevents rapid positioning at the target position. This phenomenon is thought to occur because the pneumatic pressure can not be sufficiently reduced before the particle reaches the target position, causing the high-speed movement to persist even near the target position. The electro-pneumatic regulator we used has a typical response time of 0.4 s [32]. Therefore, the air pressure cannot be reduced sufficiently if the particles reach the target position faster. The flow direction can be switched as frequently as 120 Hz, and the visual feedback is 150 Hz, which is more responsive than an electropneumatic regulator. Therefore, the electropneumatic regulator is expected to act as the limiting factor. Consequently, a response time of approximately 0.5 s at which positioning succeeds without vibration is considered the upper limit of the achievable response time for the system. Compared with piezoelectric device-driven syringe pumps [9], whose responsivity is 15 ms, our system is slow. However, pneumatic actuators are more economical than piezoelectric devices, and an appropriate selection, depending on the required specifications, is effective.

### 4.2. Shortcomings of the Particle Detection Process

**Figure 16:**
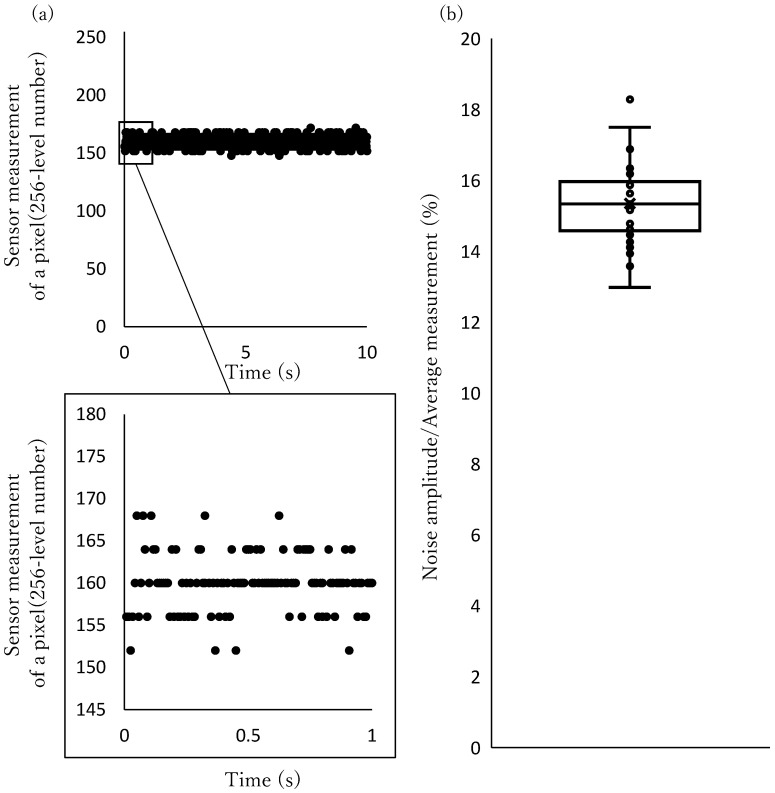
Camera noise: (**a**) Sensor measurement of a pixel. (**b**) Noise amplitude ratio to average value.

Because the particles are detected with a change in the brightness of the image captured by the camera, the detection process is vulnerable to malfunctions caused by illumination drift and camera noise. Sensor measurement of a pixel is shown in Figure 16a. As shown in this figure, sensor measurement exhibits noise. The measurements of ratio of the noise to the average value of 30 pixels were shown on a box-and-whisker diagram of Figure 16b. The typical camera noise amplitude was about 15% of the average value.

Irrelevant fluctuations in the image sensor values with a certain amplitude can be ignored by setting a threshold in exchange for sensitivity. To achieve versatility, a sensitive and robust detection process is required.

Furthermore, in the presence of multiple particles or debris within the camera field of view, this system can fail to limit its control to a single particle. Therefore, an algorithm that ignores objects other than single particles is required.

### 4.3. Compensation for Disturbance with Integral Control

**Figure 17:**
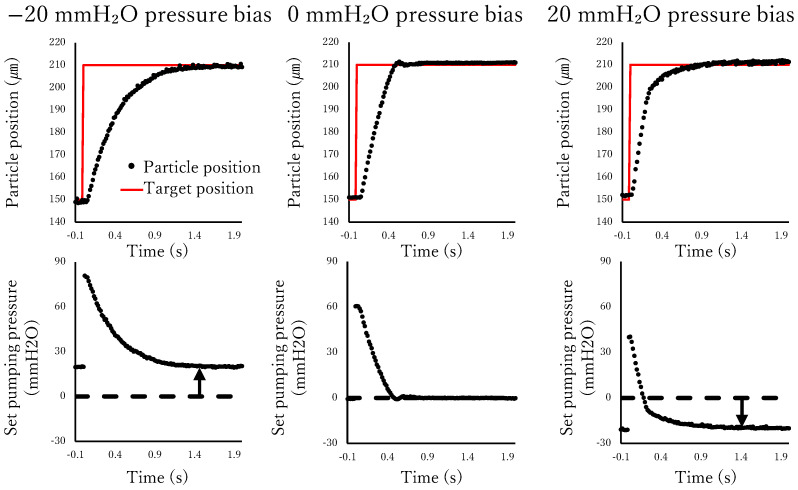
Positioning response under different pressure bias.

To normalize the experimental conditions, external pressure bias on the flow path was eliminated by connecting both ends of the flow path to beakers with leveled water surfaces. However, in practical applications, the pressures at both ends of the flow path may be uneven. Integral control was included in the control algorithm to compensate for static pressure disturbances. The positioning responses and pumping pressures at different pressure biases are shown in Figure 17. Although these responses appear to be similar, the set pumping pressures are offset to compensate for the pressure bias. Consequently, the pumping pressure after the particle moves near the target position is not zero in the presence of a pressure bias, which is the effect of the integral control.

### 4.4. Determination of Approximated Function of Pneumatic Pressure Supply (Equation (3))

It is necessary to determine the algorithm for supplying pneumatic pressure to the pump so that the pumping pressure is proportional to the control input. However, doing this theoretically is difficult because of the nonlinearity between the elastomer membrane deformation and the pressure applied. Therefore, the function used to estimate the relationship between pneumatic pressure and pumping pressure was necessarily determined by a trial-and-error manner. Based on the experimental data, we assumed that the relationship could be approximated using a combination of linear and step-like functions. Although the function we determined has yet to be proven to be optimal compared to other approaches, it is sufficiently precise to operate the system stably. In the phase of practical applications, it can be expected that the proposed micropump can be adopted to various experiments, including the positioning of floating cells and continuously keeping microorganisms actively swimming in view. The required specifications, such as channel dimensions, pump flow rate, and positioning resolution, vary depending on the purpose. Adjusting the pump dimensions to optimize the system for its purpose is effective. However, in the present state of affairs, it is necessary to examine the relationship between driving pneumatic pressure and pumping power for each design, which takes time. Therefore, the method to derive the algorithm of pneumatic pressure supply determination directly and theoretically from the designed dimensions is expected to be effective in easily designing devices tailored for specific purposes. In the future, further analysis of the pump will be conducted to establish this method. To do this, it is necessary to consider intermediate characteristics such as the rigidity of PDMS, inertia, and viscosity of water. Therefore, the development of such a method may require numerical analysis, including CFD or FEM, as well as more experimental data.

## 5. Conclusions

In this study, we conducted experiments using a high-speed camera to provide feedback on the particle position within a microchannel and then moved the particle to a target position using a sidewall-driven micropump. By setting the proportional constant of the pump dispensing pressure appropriately relative to the position of a particle, the particle was successfully positioned within 1.5 μ
m
 of the target position in approximately 0.5 s
. Moving forward, we plan to make improvements to the system to achieve a faster positioning response. Furthermore, we are going to conduct experiments using cells to prove biological relevance. In addition, further analysis of the pump will be conducted to establish the method to derive the algorithm of pneumatic pressure supply determination directly and theoretically from the designed dimensions. 

## Figures and Tables

**Table 1:** Comparison between piezoelectric-device-driven syringe pumps and peristaltic pumps.

Actuator	Pulsation	Experiment Duration
Piezoelectric-device-driven syringe pumps	No pulsation	Limited
Peristaltic pumps	Exhibit pulsation	Indefinite

**Table 2:** Definition of the variables appeared on Equation (1).

Symbol	Unit	Description	Value
x C o G	μ m	The measurement of the center of gravity position	
*D*	pix / μ m	The resolution of the image	D = 640 pixels 300 μ m
i C o G	pix	The position of the center of gravity on digital image	i C o G = D × x C o G
( i , j )	pix	The position of a pixel on digital image	Both i and j are integers .
w ( i , j )	-	The weight on the center of gravity calculation	w ( i , j ) = 1 ( If the pixel on ( i , j ) is “ The particle ” ) 0 ( If the pixel on ( i , j ) is “ The background ” )

**Table 3:** Definition of the variables used for evaluating step response.

Symbol	Unit	Description	Value
*x*	μ m	The center of gravity position of a particle	
x t a r g e t	μ m	Target position	
t i	s	Measured time for each sampled data (with step input time set to 0)	t = t 1 , t 2 , t 3 …
ϵ	μ m	Particle position error from the target position	ϵ = x − x t a r g e t
τ	s	Positioning time (until final entry into target position ± 1.5 μ m )	τ = max { t i | ϵ ( t i ) ≥ 1.5 μ m }
ϵ O	μ m	Overshoot	ϵ O = max { ϵ ( t i ) }
ϵ ¯ ( 0.5 s )	μ m	Mean error on time 0.5 s ≤ t i ≤ 20.0 s	ϵ ¯ ( 0.5 s ) = ∑ { ϵ ( t i ) | 0.5 s ≤ t i ≤ 20.0 s } { t i | 0.5 s ≤ t i ≤ 20.0 s }
ϵ m a x ( 0.5 s )	μ m	Maximum absolute value of error on time 0.5 s ≤ t i ≤ 20.0 s	ϵ ( 0.5 s ) = max { ϵ ( t i ) | 0.5 s ≤ t i ≤ 20.0 s }
ϵ m a x ( 2.0 s )	μ m	Maximum absolute value of error on time 2.0 s ≤ t i ≤ 20.0 s	ϵ ( 0.5 s ) = max { ϵ ( t i ) | 2.0 s ≤ t i ≤ 20.0 s }
ϵ R M S E ( 0.5 s )	μ m	Root Mean Square Error on time 0.5 s ≤ t i ≤ 20.0 s	ϵ R M S E ( 0.5 s ) = ∑ { ϵ 2 ( t i ) | 0.5 s ≤ t i ≤ 20.0 s } { 0.5 s ≤ t i ≤ 20.0 s }
ϵ S D ( 0.5 s )	μ m	Standard Deviation on time 0.5 s ≤ t i ≤ 20.0 s	ϵ S D ( 0.5 s ) = ∑ { ϵ ( t i ) − ϵ ¯ 2 | 0.5 s ≤ t i ≤ 20.0 s } { 0.5 s ≤ t i ≤ 20.0 s }

## Data Availability

The datasets used and/or analysed during the current study are available from the corresponding author on reasonable request.
